# Supplementary material for: Differential Disruption of Glucose and Lipid Metabolism Induced by Phthalates in Human Hepatocytes and White Adipocytes
Source: Toxics. 2024 Mar 14;12(3):214. doi: 10.3390/toxics12030214 (PMC10974754; doi:10.3390/toxics12030214)
Supplement: Supplementary file 1 [file toxics-12-00214-s001.zip › toxics-2842729-supplementary.pdf]

Supplementary materials

for

Differential Disruption of Glucose and Lipid Metabolism Induced by Phthalates in Human Hepatocytes and White Adipocytes

Yaru Tian [1,2], Miao Xu [3], Hailin Shang [4], Lijuan You [5], Jing Yang [5], Xudong Jia [2], Hui Yang [2], Yongning Wu [2,*], Xingfen Yang [1,*] and Yi Wan [4]

[1] Food Safety and Health Research Center, School of Public Health, Southern Medical University, Guangzhou 510515, China; tianyarusmile@163.com
[2] NHC Key Laboratory of Food Safety Risk Assessment, China National Center for Food Safety Risk Assessment, Beijing 100021, China; jiaxudong@cfsa.net.cn (X.J.); yanghui@cfsa.net.cn (H.Y.)
[3] Department of Clinical Nutrition, West China Hospital, Sichuan University, Chengdu 610044, China; xumiao1726@wchscu.cn
[4] Laboratory for Earth Surface Processes, College of Urban and Environmental Sciences, Peking University, Beijing 100871, China; shanghailin@stu.pku.edu.cn (H.S.); wany@urban.pku.edu.cn (Y.W.)
[5] School of Public Health, Shandong Second Medical Universtiy, Weifang 261053, China; 20220846@stu.wfmc.edu.cn (L.Y.); 20220848@stu.wfmc.edu.cn (J.Y.)
* Correspondence: wuyongning@cfsa.net.cn (Y.W.); xfyang@vip.163.com (X.Y.)

Chemicals Information

Phthalate	Abbreviation	CAS number	Cat number
Di(2-ethylhexyl) phthalate	DEHP	117-81-7	ALR-097N
Monoethylhexyl phthalate	MEHP	4376-20-9	ALR-138N
Dicyclohexyl phthalate	DCHP	84-61-7	ALR-099N
Monocyclohexyl phthalate	MCHP	7517-36-4	ALR-178N

Table S1. Chemicals Information

Regent Information

Regent	CAS number	Cat number	Brand
Calcium pantothenate	137-08-6	C8731-25G	Sigma Aldrich
Human insulin	11061-68-0	HY-P0035	MCE
Biotin	58-85-5	B4639-100mg	Sigma Aldrich
Dexamethasone	50-02-2	D4902-25mg	Sigma Aldrich
Indomethacin	53-86-1	I7378-10G	Sigma Aldrich
Isobutylmethylxanthine	28822-58-4	I5879-100mg	Sigma Aldrich
3,3′,5-Triiodo-L-thyronine	6893-02-3	T2877-100MG	Sigma Aldrich
GW6471	880635-03-0	HY-15372	MCE
GSK3787	188591-46-0	HY-15577	MCE
T0070907	313516-66-4	2301	R&D
Rosiglitazone	122320-73-4	71740	Cayman

Table S2. Regent Information

AMPK signaling pathway

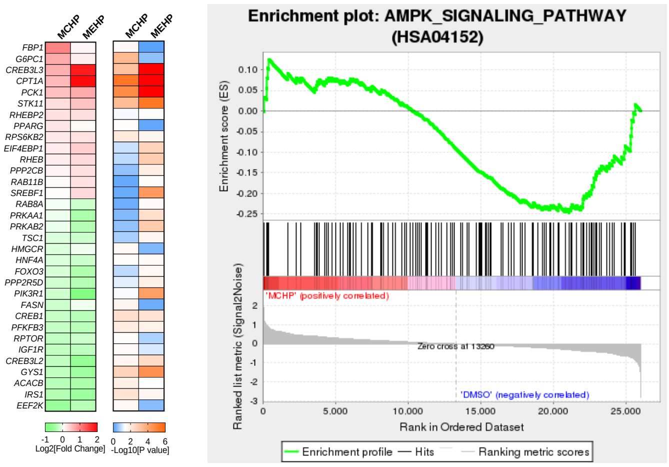

Figure S1. DEGs and GSEA of AMPK signaling pathway.

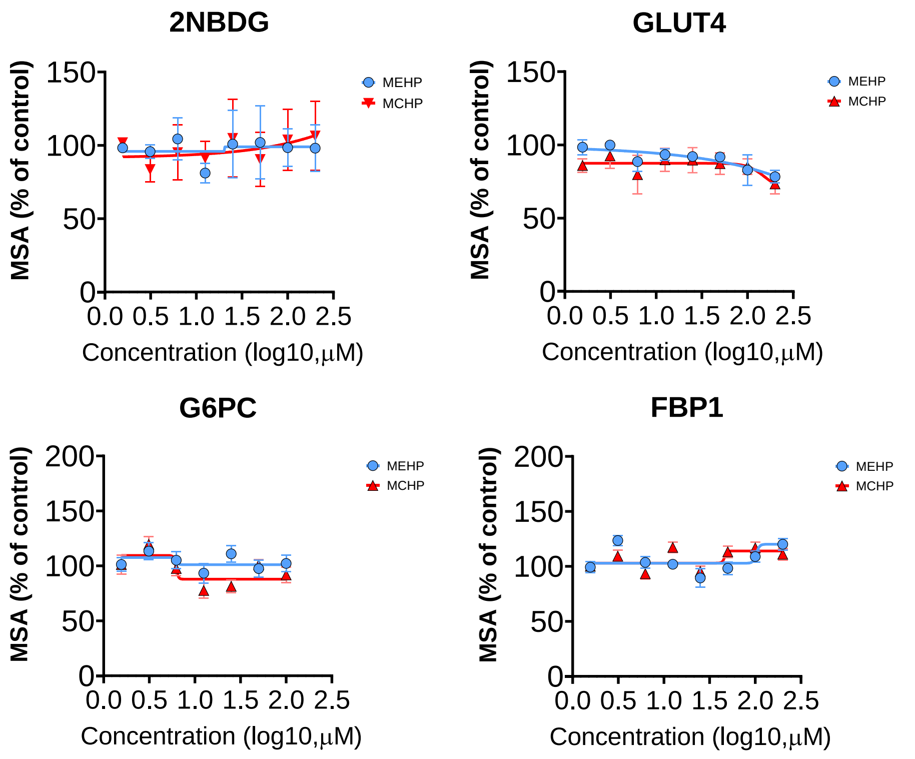

Figure S2. The effects of MEHP and MCHP on glucose metabolism in MA cells.
